# Two novel strategies to overcome the resistance to ALK tyrosine kinase inhibitor drugs: Macrocyclic inhibitors and proteolysis‐targeting chimeras

**DOI:** 10.1002/mco2.42

**Published:** 2021-06-17

**Authors:** Xiaoling Song, Hui Zhong, Xiaojuan Qu, Linsong Yang, Biao Jiang

**Affiliations:** ^1^ Shanghai institute for advanced immunochemical studies ShanghaiTech University Shanghai China; ^2^ School of pharmaceutical engineering and life science Changzhou University Jiangsu China; ^3^ Chinese academy of sciences Shanghai institute of organic chemistry Shanghai China

**Keywords:** ALK PROTACs, ALK‐TKIs, EML4‐ALK, macrocyclic inhibitors, non‐small cell lung cancer, resistance mechanisms

## Abstract

Lung cancer is the most malignant tumor in the worldwide. About 3%‐5% non‐small cell lung cancer (NSCLC) patients carry anaplastic lymphoma kinase (ALK) gene fusions and receive great benefits from ALK‐targeted therapy. However, drug resistance inevitably occurs even with the most potent inhibitor drug lorlatinib. About half of the resistance are caused by alteration in ALK proteins for earlier ALK TKI drugs and near one‐third of loratinib resistant cases are caused by compound mutations without current effective treatment strategy in clinic. Novel strategies are in great need to overcome drug resistance. Lately, two novel strategies have been developed and attracted great attentions for their potentials to overcome drug resistance problems: (1) developed small compact macrocyclic ALK kinase inhibitors and (2) developed ALK targeted proteolysis‐targeting chimera (PROTAC) drugs. The macrocyclic molecules are small and compact in size, brain barrier permeable, and highly potent against lorlatinib‐resistant compound mutations. Developed ALK targeted PROTAC molecules could degrade oncogenic ALK driver proteins. Some showed superiority in killing ALK positive cancer cells and inhibiting the growth of cells expressing G1202R resistant ALK proteins comparing to inhibitor drugs. The update on these two treatment strategies was reviewed.

## Graphical abstract

Acquired resistance mutations including G1202R and L1196M, alone or in compound form, in ALK proteins lead to drug resistance to ALK‐targeted therapy. Two novel strategies showed great potential in overcoming such resistance: (1) More effective inhibition by small and compact macrocyclic inhibitors; (2) Degradation of oncogenic ALK proteins including those with resistant mutations by ALK targeted proteolysis‐targeting chimeras.

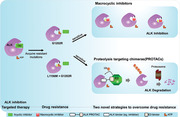

**Abbreviations**
ALK: anaplastic lymphoma kinase
CDX: cell line‐derived xenograft
CRBN: cereblon
FDA: food and drug administration
IC50: half maximal inhibitory concentration
IND: investigational new drug
NSCLC: non‐small cell lung cancer
NTRK: neurotrophin receptor kinase
PDX: patient‐derived xenograft
PROTAC: proteolysis targeting chimera
ROS1: ROS proto‐oncogene 1
TBK1: TANK binding kinase 1
TKIs: tyrosine kinase inhibitors
VHL: von Hippel‐Lindau

## 1 INTRODUCTION

Lung cancer is the most common malignant cancer in the world, and it is also the leading cause of cancer‐related death. The latest data published by the national cancer center in 2020 showed that in 2015 lung cancer accounted for 20% of new cases in China and led to 27% of cancer deaths, ranking first in both morbidity and mortality among malignant tumors in China.^1^ A latest report released that lung cancer has the highest fatality rate of malignant tumor in the United States, and the 5‐year lung Cancer survival rate is 18.4%.^2^ Treating ALK mutant cancer patients with targeted therapy greatly reduces toxic adverse effect and improves patients’ quality of life comparing to traditional chemotherapy.^3^

**FIGURE 1:**
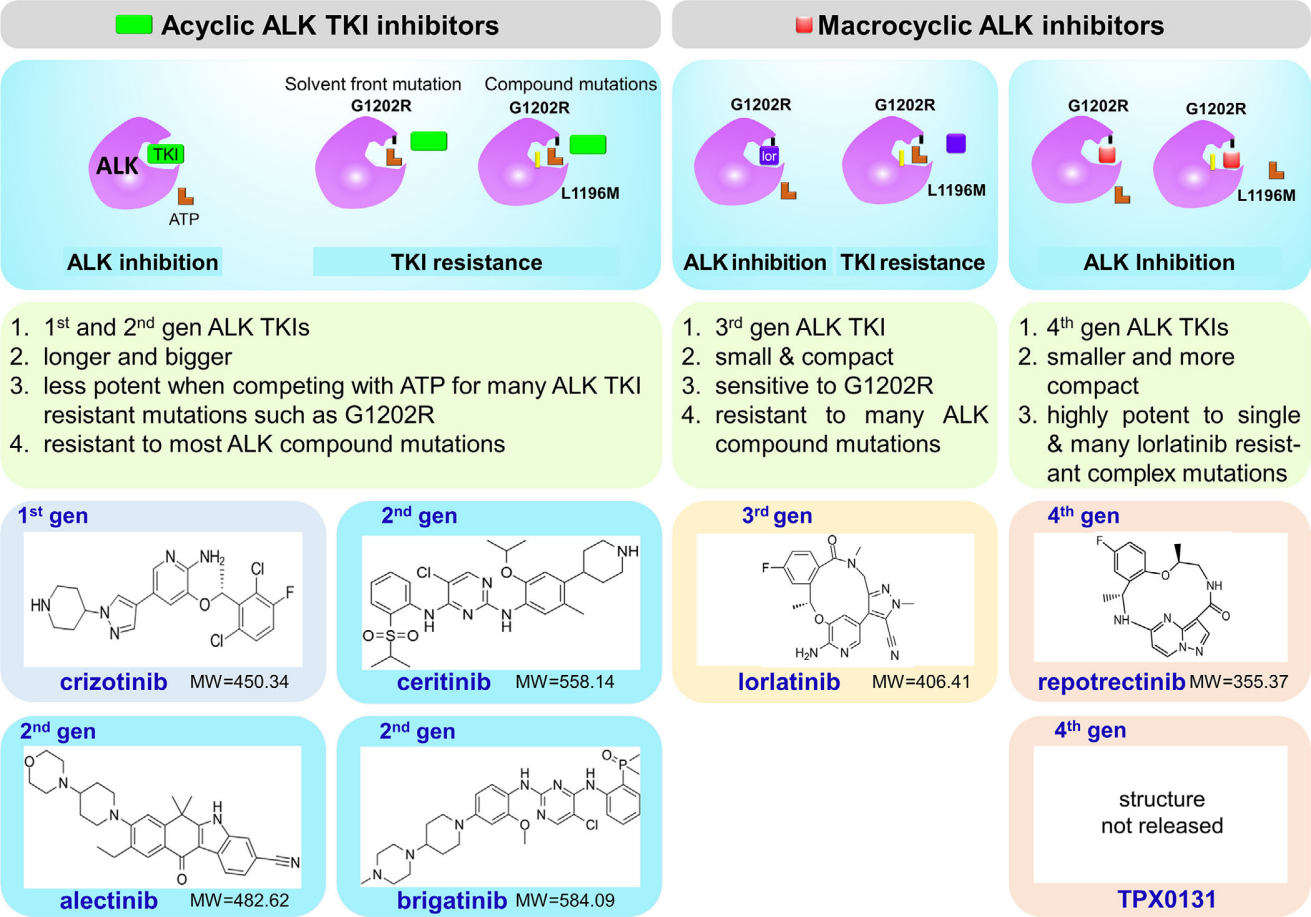
FDA approved ALK inhibitor drugs and novel macrocyclic ALK inhibitors. The structure and molecular weight (MW) of five FDA approved ALK inhibitor drugs (1st to 3rd generations) and newly developed macrocyclic ALK kinase inhibitors were presented. When there are no resistant mutations in ALK tyrosine kinase domain, ALK kinase activities can be effectively inhibited by all currently approved ALK tyrosine kinase inhibitor drugs (TKIs). The 2nd generation ALK TKI drugs can effectively inhibit the activity of gate keeper mutation L1196M, but they are not so effective on solvent front mutation G1202R and many compound mutations. The 3rd generation ALK TKI drug lorlatinib is a small and compact macrocyclic inhibitor. It can effectively inhibit the activity of G1202R mutation but not for those compound mutations. Latest developed macrocyclic ALK TKI drugs are even smaller and more compact than lorlatinib. They are highly potent against G1202R mutation and many lorlatinib resistant compound mutations. They were proposed to be the next generation ALK TKI drugs (references 14, 19) Abbreviations: MW, molecular weight.

At present, five different ALK‐Tyrosine kinase inhibitors (ALK‐TKIs) have been approved by food and drug administration (FDA) to treat ALK‐positive non‐small cell lung cancer (NSCLC) patients (Figure 1). These drugs include the first‐generation ALK‐TKIs crizotinib, the second‐generation ALK‐TKIs ceritinib, alectinib and brigatinib, and the third generation ALK‐TKIs lorlatinib. Although later generation ALK TKI inhibitors have better kinase selectivity and enhanced ability to overcome drug resistance, it has been proved that drug resistance inevitably occurs within a certain period of time after the initial drug treatment. It is urgent to develop novel and effective treatment strategies to overcome drug resistance. This article was focused on the update on two novel strategies to overcome drug resistance.

## 2 RESISTANCE MECHANISMS OF ALK KINASE INHIBITORS

About half of the acquired resistance mechanisms to ALK TKIs are ALK kinase protein‐dependent. The acquired resistance mechanisms of first‐generation and second‐ generation ALK‐TKIs include: secondary mutations in ALK kinase domain, amplification of ALK fusion gene, epithelial mesenchymal transformation, activation of ERK, SRC, IGF‐1R and other bypass signaling pathway, and transformation into small‐cell lung cancer.^4^ Firstly, within above resistant mechanisms, ALK‐dependent resistant mechanisms including ALK gene amplification and acquired secondary mutations in ALK kinase domains make up about half of the incidence for resistance to crizotinib (1st generation ALK TKI).^5^, ^6^ These mutations include L1196M, L1152R, and G1202R, etc. Because drug‐resistant mutations transform ALK protein into an active conformation and introduce new steric hindrance between ALK kinase and ATP, they increase the binding affinity between the kinase and ATP resulting in drug resistance. Secondly, secondary mutations in the ALK kinase domain are more common in patients with acquired resistance to later generation ALK inhibitor drugs, reaching about 50‐70% of resistant cases.^7^ The mutation spectrum was also different from that of crizotinib. Solvent front mutation G1202R is the most dominant acquired mutation among mutations that confer the resistance to second generations ALK TKI drugs. It makes up about 42% of resistant cases for patients on brigatinib treatment, 21% for patients treated with ceritinib, and 29% for patients on alectinib.^7^ These results indicate that acquired aberrant ALK protein alterations are the major source of drug resistance. Developing strategies to target these aberrant ALK alterations are critical to overcome such drug resistance.

The third generation ALK TKI drug lorlatinib is a potent, brain permeable, small macrocyclic molecule (Figure 1). It can inhibit most of the drug‐resistant mutations of crizotinib and second‐generation ALK‐TKIs especially G1202R mutation.^8^ Clinical data showed that lorlatinib led to an objective response rate of 45% and durable responses in ALK‐positive patients who already developed resistance to previous generation of ALK TKI drugs.^9^, ^10^ Lorlatinib was approved by FDA in 2018 for second‐line treatment of advanced ALK‐positive NSCLC patients. A full user guide to lorlatinib has been recently reviewed.^11^

However, the development of drug resistance to lorlatinib is also inevitable in clinic. Studies showed that about 35% of the acquired resistance mechanisms to lorlatinib are compound mutations at two or three sites, such as I1171N + L1198F, G1202R + L1204V + G1269A (Table 1).^12^ In addition, other complex mutations conferring drug resistance to lorlatinib were also reported.^13^ The sensitivities of 14 different ALK compound mutations that conferring resistance to lorlatinib have been studied using current FAD approved ALK inhibitor drugs and were summarized in Table 1. Results showed that some of the compound mutations (6/14) were sensitive to 1st or 2nd generation of ALK TKI inhibitors, but about half of compound mutations were resistant to all current approved ALK TKI drugs. Such resistant mutations include ALK G1202R+L1196M, G1202R+F1174C, and G1202R+ F1174L etc. Therefore, novel treatment strategies are in great need to be developed to solve these treatment difficulties in clinic.

**TABLE 1:** Reported mutations resistant to lorlatinib and their sensitivities to other ALK kinase inhibitors

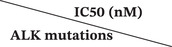	**lorlatinib 3^rd^**	**crizotinib 1^st^**	**ceritinib 2^nd^**	**alectinib 2^nd^**	**brigatinib 2^nd^**	**repotrectinib**	**TPX‐0131**	**Reference**
**Lorlatinib sensitive variants**								
WT	0.7‐1.9	22‐74.8	2.1‐10	10‐18.9	2.4‐11.8	17.8	<5	13,15,19
G1202R	69‐80	230‐560	120‐388	507‐640	56‐399	20.5	<2	13,15,19
L1196M	18	713	6	131	13	50	–	13
I1171N	81	220	34‐36	600	27‐28	–	–	13
**Lorlatinib resistant variants**								
C1156Y + L1198F	67‐140	0.6‐22	1123‐1493	24‐776	12‐118	1.1	–	12,13,15,35
L1196M + L1198F	1169	350.7	1794	1249	341	34.8	<2	15,19
G1202R + L1198F	132‐370	47	477‐1100	>1000	290‐2040	0.2	<2	12,13,15,19
G1202R + C1156Y	>1000	>1000	>1000	>1000	>1000	–	<2	13,19
G1202R + G1269A	700	390	120	960	75	–	–	12,13
G1202R + L1196M	>1000	370‐800	690‐1260	>1000	320‐1100	–	<2	12,13,19
G1202R + F1174C	280‐300	320‐370	280‐420	700‐1400	180‐220	–	–	13
G1202R + F1174L	110‐190	200‐370	260‐420	560‐2300	160‐190	–	–	13
G1202R + L1204V + G1269A	673	634	345	6740	176	–	–	12,13
I1171N + L1198F	240	19	660	750	43	–	–	12,13
I1171N + L1196M	360	460	21	460	31	–	–	12,13
I1171N + L1256F	6000	560	400	73	42	–	–	12,13
I1171N + G1269A	470	670	14	1100	7.1	–	–	12,13
I1171N + L1198H	670	160	540	1600	140	–	–	13
I1171N + F1174I	320	350	140	1300	110	–	–	13
I1171N + F1174L	220	440	160	1600	100	–	–	13
I1171N + D1203N	Resistant	–	–	–	–	–	–	12
E1210K + D1203N + G1269A	Resistant	–	–	–	–	–	–	12

Half maximal inhibitory concentration (IC50s) in Ba/F3 cells for different ALK TKI inhibitors were summarized. Lorlatinib resistant mutations reported in clinics or identified in lab were labeled in bold color.

– : means data are not available.

## 3 NOVEL TREATMENT STRATEGIES

Lots of efforts have been put to develop newer treatment strategies to fight against drug resistance. Two latest developed treatment strategies are as follows: (1) developing smaller highly compact macrocyclic ALK‐TKIs and (2) developing ALK‐targeting proteolysis targeting chimeria (PROTAC) molecules. The updates on these treatment strategies were reviewed in the following.

### 3.1 Develop smaller and more compact macrocyclic ALK‐TKIs

Most FDA approved ALK‐TKIs are ATP‐competitive type I TKIs, which are large in size and have some motifs close to or across the hydrophobic posterior capsule, which makes them susceptible to various drug‐resistant mutations^14^ (Figure 1). Further development of similar inhibitors is difficult to block the activities of such evolutionary mutations. Based on this idea, new inhibitors were designed smaller and more compact to avoid previous resistance problems. For example, the third generation lorlatinib was designed as a small macrocyclic molecule (Figure 1). It is smaller than all earlier generation of FDA approved ALK inhibitor drugs; and it is more potent in inhibiting the activities of mutant ALK kinase proteins than all previous approved ALK TKI drugs. Therefore, newer ALK TKIs were designed even smaller and more compact to evade the intrinsic resistance to previous reported mutations. Two new small and compact macrocyclic molecules were designed by Turning Point Therapeutics, Inc. lately, which are repotrectinib and TPX‐0131. The molecular weight of these two molecules was smaller than all current FDA approved ALK TKI drugs (Figure 1). Because these two molecules are very potent against many lorlatinib resistant compound mutations, they are proposed to be the 4th generation ALK TKI drugs.

Repotrectinib (TPX‐0005) is one of the two small compact macrocyclic molecules (Figure 1). It is smaller than lorlatinib with a molecular weight about 355 Kilo‐Dalton and having a high activity in central nervous system.^14^ It can accurately anchor molecules on adenine binding sites on ALK protein, target kinase resistant active conformations, effectively avoid mutations especially G1202R precursor solvent mutation caused by space interference.^14^ Data showed that repotrectinib could effectively inhibit previously reported tough resistant mutations including G1202R and L1196M, and it is also effective in some lorlatinib resistant ALK compound mutations such as L1198F+C1156Y and G1202R+L1198F (Table 1).^15^ Additionally, It effectively induced G1202R resistant tumor regression in clinical relevant mouse models including both cell line derived xenograft (CDX) and patient‐derived (PDX) tumor models.^16^ Furthermore, repotrectinib can also sensitize H2228 cells that are intrinsically resistant to ALK TKI by inhibiting SRC activities.^17^, ^18^ Lastly, it is a multi‐function inhibitor that can inhibit the activity of NTRK (neurotrophin receptor kinase), ROS1 (ROS proto‐oncogene 1), and ALK.^14^ In general, repotrectinib is a very promising treatment to overcome various mechanisms of ALK resistance, including secondary mutations and bypass signal activation. The efficacy of repotrectinib in patients with advanced solid tumor harboring ALK gene rearrangement is currently being evaluated in a phase I/II clinical trial (www.clinicaltrials.gov: NCT03093116).

TPX‐0131 is another small compact macrocyclic ALK inhibitor developed by Turning Point Therapeutics. It was firstly reported in 2020 AACR annual meeting. Released data showed that TPX‐0131 is not only sensitive to most single resistant mutations including solvent front G1202R resistant mutation, but also is very effective for many compound mutations such as G1202R+L1196M, G1202R+L1198F, G1202R+C1156F, and L1196M+ L1198F.^19^ Currently, this compound is in a process of investigational new drug enabling study. With its strong activity in overcoming many lorlatinib resistant compound mutations, TPX‐0131 has the great potential to be the candidate of the next‐generation ALK inhibitor drug.

### 3.2 Proteolysis targeting chimeras of ALK

**FIGURE 2:**
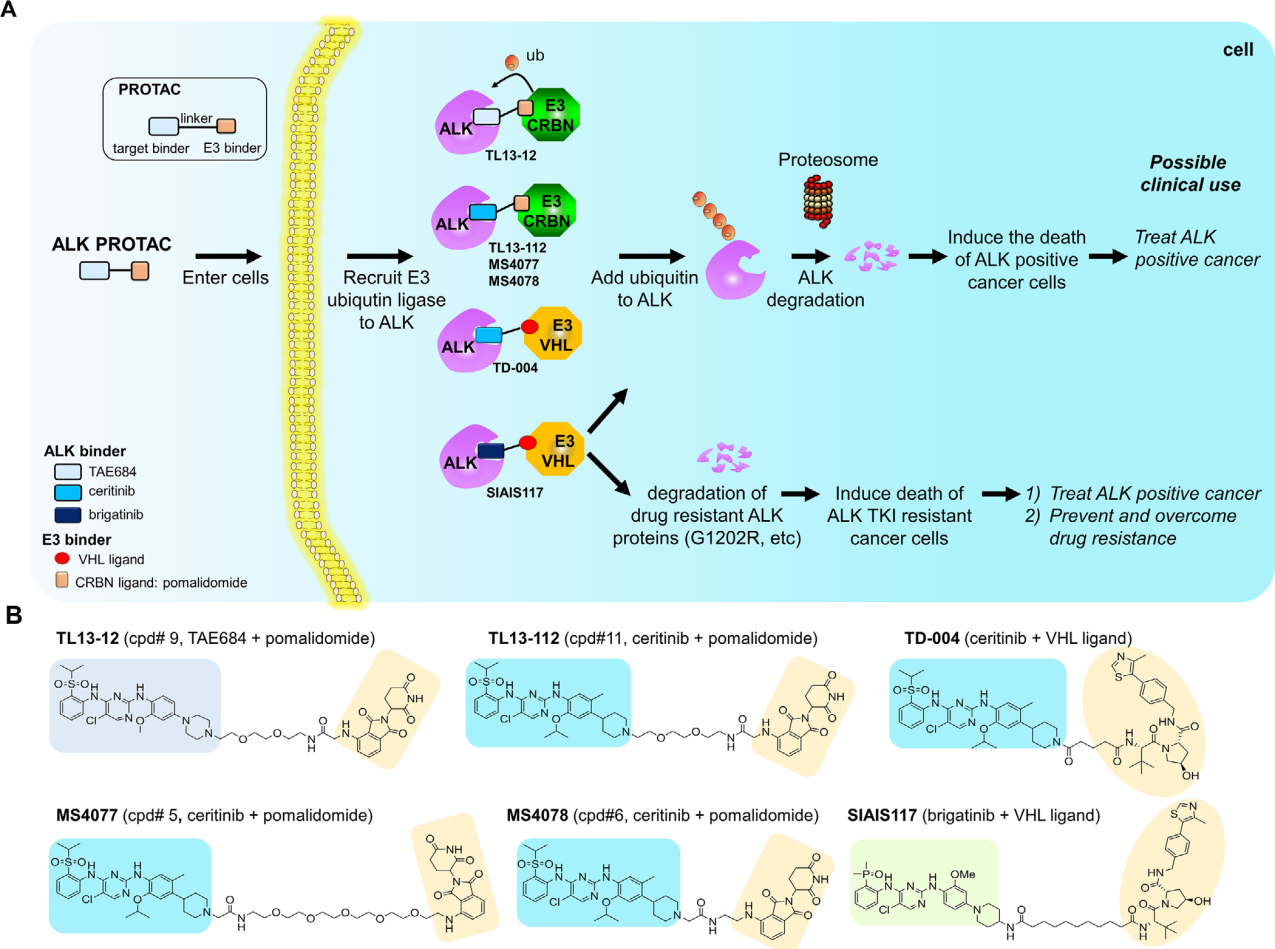
ALK‐targeted proteolysis targeting chimeras. A, The working principles of ALK PROTAC molecules. An ALK‐PROTAC molecule includes three parts: (1) an ALK protein binder, (2) an E3 ubiquitin ligase binder, and (3) a connection linker. When an ALK PROTAC molecule enters cells, it binds to ALK protein and in the meanwhile, it recruits the E3 ubiquitin ligase to the proximity of ALK protein. Followed by labeled with ubiquitin, ALK protein was degraded by proteasome. The degradation of ALK protein by ALK PROTACs will lead to the death of ALK positive cancer cells, including those baring TKI resistant mutations, provide a promising strategy to treat cancer patients and overcome drug resistance in clinic in the future. B, Six published ALK PROTAC molecules. Three different ALK binders were used: TAE684, ceritinib, and brigatinib (as shown in left rectangular part); and two different E3 binders were used: pomalidomide and VHL ligand (as shown in brown colored right part)

PROTAC technology is a technology utilizing cell endogenous proteasome degradation system to degrade protein of interest. The compounds designed based on PROTAC technology are hetero‐functional molecules (Figure 2A), which include three parts: (1) ligand for targeted protein, (2) ligand to recruit an E3 ubiquitin ligase, and (3) a linker for connection of the two ligands. The most frequently used E3 ubiquitin ligase ligands include cereblon (CRBN) ligands and VHL (von‐Hippel‐Lindau) ligands. CRBN ligands include thalidomide, lenalidomide, and pomalidomide. Both CRBN protein and VHL protein are substrate recognition subunits of two widely expressed and functionally important cullin ring E3 ubiquitin ligases complexes. By connecting the target protein and the E3 ligase together, PROTAC molecules recruit the target protein to the proximity of the E3 ubiquitin ligase, which leads to ubiquitination of the target protein, and subsequently degradation of target protein by proteasome. Because aberrant expression of many biologically important proteins are highly involved in the progress of human diseases, targeted degradation of these proteins have been considered as a critical way to treat such diseases. Because of its great potential to degrade previously undrugable protein and overcome drug resistance in precision medicine, it has attracted a lot of attentions recently to be used in the development of new drugs for human diseases.^20^ At present, PROTAC technique has been successfully applied to selective degradation of various protein targets, such as BCR‐ABL,^21^ TBK1,^22^ epidermal growth factor receptor (EGFR),^23^ HER2,^23^ and c‐MET.^23^

Because oncogenic ALK protein is the driver force of ALK mutant positive cancer, and about half of the resistant mechanisms are related to either ALK mutations or ALK amplification. Targeted degradation of ALK protein theoretically can improve drug treatment efficacy and evade drug resistance. In addition, although ALK is expressed in many types of cancer, it is barely expressed in adult animals.^24^ Get rid of ALK protein in patients will not affect normal function of human health and only affect ALK dependent tumors. Furthermore, mouse models have showed that knock out ALK only had a slight impact on the behavioral phenotype of mice without affecting their survival.^25^ Therefore, pharmacological degradation of ALK by PROTAC technology can effectively kill cancer cells, reduce the off‐target side toxicity effect and provide a new and potential treatment strategy for ALK‐positive cancer patients.

Currently, there are six different ALK PROTACs having been developed (Figure 2 and Table 2). All published ALK PROTACs were designed based on the second generation ALK‐TKIs. Based on the ALK binder, these ALK targeting PROTACs can be categorized into three classes: (1) ceritinib‐based ALK PROTAC^26^, ^27^, ^28^, ^29^, (2) TAE684‐based ALK PROTAC^27^, and (3) brigtinib‐based ALK PROTAC.^30^

**TABLE 2:** Bio‐activities of reported ALK PROTAC molecules

ALK PROTACs	TL13‐12	TL13‐112	MS4077	MS4078	TD‐004	SIAIS117
**PROTAC elements**						
ALK binders	TAE684	Ceritinib	Ceritinib	Ceritinib	Ceritinib	Brigatinib
E3 type	CRBN	CRBN	CRBN	CRBN	VHL	VHL
E3 binders	Pomalidomide	Pomalidomide	Pomalidomide	Pomalidomide	VHL ligand	VHL ligand
**In vitro DC50 (nM)**						
EML4‐ALK (NSCLC cell line)	10 (H3122)	10 (H3122)	34 (H2228)	59 (H2228)	300 (H3122)	∼100 (H2228)
NPM‐ALK (ALCL cell line)	180 (Karpas299)	40 (Karpas299)	3 (SUDHL1)	11 (SUDHL1)	300 (SUDHL1)	7 (SR)
ALK ^F1174L^ (NB cell line)	50 (Kelly)	50 (Kelly)	–	–	–	–
EML4‐ALK^G1202R^	–	–	–	–	–	189
**In vitro IC50 (nM)**						
EML4‐ALK	25	50	30	45	180	46
NPM‐ALK	5	5‐15	46	33	58	1.7
EML4‐ALK^G1202R^	–	–	–	–	–	166
Activity (comparing to inhibitor)	Slightly stronger	Slightly stronger	Weaker	Weaker	Weaker	Slightly stronger
Effect on bypass pathway	–	–	–	–	–	Inhibit SCLC
Degrade Aurora A	Yes	Yes	–	–	–	No
**In vivo activities**						
Serum levels	–	–	–	>500 nM (IP, 50 mg/kg)	–	–
Anti‐tumor activity	–	–	–	–	Inhibit H3122 CDX growth (IP)	–
**Reference**	27	27	28	28	26	30

Abbreviations: CDX, cell line‐derived xenograft; DC50, half maximal degradation concentration; IC50, half maximal inhibitory concentration; IP, intraperitoneal drug administration.

– : No data available.

Ceritinib‐based ALK PROTACs exhibited a moderate ALK protein degradation ability. In these studies, ligands for two different E3 ubiquitin ligases, CRBN (cereblon), and VHL (von Hippel‐Lindau), were linked to ceritinib to generate ALK PROTAC molecules (Figure 2B and Table 2). ALK PROTACs using polidomide as CRBN E3 ligase ligand exhibited stronger degradation ability comparing to those using VHL ligand (Figure 2B and Table 2). Kang et al^26^ used VHL as E3 ligands to generate ALK degrader TD‐004. At the concentration of 300 nM, TD‐004 could degrade NPM‐ALK fusion protein in ALCL cell line SU‐DHL‐1 cells and degrade EML4‐ALK protein (Version 1) in non‐small cell lung cancer cell line H3122. Powell et al^27^ developed ALK degraders with CRBN ligand pomalidomide. ALK degrader TL13‐112 degraded NPM‐ALK protein at the concentration of 40 nM in ALCL cell line Karpas299 and degraded EM4‐ALK at the concentration of 10 nM in H3122 cell lines. Zhang et al also utilized CRBN ligand pomalidomide to design their ALK degrader, which could degrade NPM‐ALK (SU‐DHL‐1) and EML4‐ALK (version3, in non‐small cell lung cancer cell line H2228) at the concentration of 3‐60 nM.^28^ Besides using ceritinib in ALK PROTAC, Powell et al also used TAE684 and pomalidomide to generate ALK PROTAC molecules, but the degradation capability of TAE684 PROTAC was not better than that of ceritinib‐pomalidomide PROTAC.^27^

However, there was no dramatic improvement for ceritinib‐based ALK degraders comparing to ceritinib in terms of anti‐proliferation effect against ALK mutant lung cancer cells (Figure 2B and Table 2). In NSCLC and ALCL cell lines, the anti‐proliferation activities of the degraders and parental kinase inhibitors are roughly the same. VHL‐based ceritinib ALK degrader TD‐004 could inhibit the proliferation of SU‐DHL‐1 but with the ability reduced about 2‐20 fold (Table 2). The ceritinib‐pomalidomid ALK degraders MS4077 and MS4078 designed by Zhang exhibited an anti‐proliferation ability (IC50s) of 33 nM and 46 nM in SU‐DHL‐1 cells, and which was about 2‐3 fold weaker than that of ceritinib (IC50: 15 nM). Ceritinib‐ and pomalidomide‐based degraders TL13‐12 and TL13‐112 designed by Powell could inhibit the proliferation of H3122, Karpas299, and SU‐DHL‐1 cells, but the IC50 was about similar to or weaker than ceritinib.^27^ They also tried to study the effect on the growth inhibition of Ba/F3 cells expressing EML4‐ALK resistant mutations. However, no ALK degrader showed better growth inhibition effect than ceritinib did.^27^

Brigatinib‐based ALK degrader displayed a good potential to overcome drug resistance (Figure 2B and Table 2). Currently, only one brigatinib‐based ALK degrader, SIAIS117, was reported, which used ligand of VHL as the E3 ubiquitinase ligand.^30^ It could not only effectively degrade NPM‐ALK proteins in ALCL cell line SR and EML4‐ALK proteins in NSCLC cell line H2228, but also could degrade exogenously expressed ALK fusion proteins with G1202R resistant mutations in 293T cells. Additionally, it also exhibited a better growth inhibition effect in several ALK mutation positive cancer cells including ALCL cell line SR, non‐small cell lung cancer cell line H2228, and ALK TKI resistant cells that exogenously expressed G1202R mutant EML4‐ALK proteins. Furthermore, SIAIS117 displayed an advantage in inhibiting the growth of two different small cell lung cancer cell lines at a physiological achievable concentration for most approved drugs, but brigatinib did not have such function at the same concentration. Cell type transformation from lung adenocarcinoma cancer cells to small cell lung cancer cells is one resistance mechanism to crizotinib.^31^, ^32^, ^33^ Thus, SIAIS117 is potentially effective in overcoming such resistance by possessing a more potent growth inhibition effect on small cell lung cancer cells.

Two different data supported that ALK PROTAC molecules could also have good bioavailabilities in vivo (Table 2). When administered ALK PROTAC molecules MS4078 in mice via intraperitoneal injection, it was pharmacokinetically stable. It could achieve a relative stable serum exposure level more than 500 nM within a 12‐hour observing window^28^ (Table 2). ALK degrader TD‐004 could inhibit the growth of H3122 xenograft tumor in mouse model after intraperitoneal administration^26^ (Table 2). A big obstacle for PROTAC molecules to become successful drug candidates in clinic is that most generated PROTAC molecules barely had good exposure in vivo. However, the data from MS4078 and TD‐004 indicate that current PROTAC developing technologies have broken the obstacle and are able to generate ALK PROTAC candidates with good pharmacokinetics in vivo. This will greatly expedite the speed of developing a successful ALK PROTAC drug to be used in clinic in the future.

Different ALK degraders have different side effects on cell activities. Ceritinib‐based ALK degraders and TAE684‐based ALK degrader showed some non‐specific degradation of cellular proteins such as Aurora A.^27^ However, brigatinib‐based ALK PROTAC SIAIS117 did not degrade Aurora A. It indicates a different ALK binder may bring in differential specificity to the final cellular action. Because brigatinib also provides patients with a longer progression‐free survival rate comparing to other second generation ALK inhibitor drugs, brigatinib‐based ALK degraders may be more beneficial than ceritinib‐based ALK degraders in treating cancer patients in the future.

## 4 CONCLUSIONS

In summary, two different strategies have been developed to overcome current drug resistance issues. Designing smaller and more compact macrocyclic ALK inhibitors seems to be a very good way to develop effective ALK targeted drug candidates. Small and compact macrocyclic can bind completely inside the ATP pocket even in the presence of solvent front mutation and gatekeeper mutation. This empower them the ability to evade the resistance to previous generation ALK TKI drugs. ALK TKI drug repotrectinib (TPX‐0005) showed great potency in overcoming ALK drug resistant mutation such as L1196M and G1202R mutations, and it is currently being tested in phase I clinical trials to treat ALK TKI resistant patients. Another small compact macrocyclic molecule TPX‐0131 has also been showed with very potent activities against most of compound mutations conferring resistance to lorlatinib and currently is in an IND‐enabling study. These macrocyclic inhibitors were proposed to be the next‐generation ALK TKI drugs^14, 19^ and they will provide great opportunities to solve some current resistance problems in ALK‐positive cancer patients in the future.

The strategy of treating ALK positive cancer with ALK‐specific PROTAC molecules provides another good opportunity to overcome drug resistance problems. In order to achieve sustained ALK inhibition, inhibitors must bind to the ALK protein for a long period of time to reach binding saturation. During this process off‐target effects and kinase mutations may occur, resulting in drug resistance, becoming a major clinical obstacle. PROTAC technology provides an ideal way to degrade ALK driver proteins to kill ALK mutant cancer cells and evade drug resistance. Currently, the PROTAC technology is still in development. Most ALK degraders have not shown in vivo anti‐tumor efficacy yet. Even brigatinib‐based ALK degrader is very promising for its ability to overcome ALK drug resistance in vitro, the pharmokinetics and dynamics of this compound remain to be studied. Results from MS4078 and TD004 showed that ALK PORTACs are able to have good exposure in vivo. It indicates that current technologies are able to break the obstacle of poor exposure for PROTAC molecules in vivo, and ALK PROTAC molecules have great opportunities to become real therapeutic drugs in clinic in the future. The latest news from ASCO2020 showed that the pioneer PROTAC drug ARV110 developed by Arvinas could induce tumor regression in cancer patients in clinical trials (NCT03888612).^34^ It proved that PROTAC molecules could have good biological effect in clinic in real patients. With the fast developing PORTAC field, more and more PROTAC drugs are anticipated to be shown in clinical trials, and the time for a successful ALK PROTAC drug to be used in clinic is about only 5‐10 years away.

## CONFLICT OF INTEREST

The authors declare no conflict of interest for the manuscript.

## AUTHOR CONTRIBUTIONS

Xiaoling Song, Hui Zhong, and Xiaojuan Qu wrote the manuscript; Xiaoling Song designed and revised the manuscript; Xiaoling Song, Biao Jiang, and Linsong Yang supervised the work.

## ETHICS STATEMENT

Not applicable.
